# Supplementary material for: Inflammation-induced IgE promotes epithelial hyperplasia and tumour growth
Source: eLife. 2020 Jan 14;9:e51862. doi: 10.7554/eLife.51862 (PMC6959995; doi:10.7554/eLife.51862)
Supplement: Transparent reporting form [file elife-51862-transrepform.pdf]

eLIFE
1st Floor
24 Hills Road
Cambridge CB2 1JP, UK
P 01223 855340
W elifesciences.org
T @elife

eLife's transparent reporting form

We encourage authors to provide detailed information *within their submission* to facilitate the interpretation and replication of experiments. Authors can upload supporting documentation to indicate the use of appropriate reporting guidelines for health-related research (see EQUATOR Network), life science research (see the BioSharing Information Resource), or the ARRIVE guidelines for reporting work involving animal research. Where applicable, authors should refer to any relevant reporting standards documents in this form.

If you have any questions, please consult our Journal Policies and/or contact us: editorial@elifesciences.org.

Sample-size estimation

- You should state whether an appropriate sample size was computed when the study was being designed
- You should state the statistical method of sample size computation and any required assumptions
- If no explicit power analysis was used, you should describe how you decided what sample (replicate) size (number) to use

Please outline where this information can be found within the submission (e.g., sections or figure legends), or explain why this information doesn't apply to your submission:

Sample sizes were chosen based on previous experience with the experimental protocols being used. In order to minimise the number of animals while detecting statistical significant differences between experimental groups, power calculations were performed, based on our extensive experience using the skin models presented. The 'power' of the experiments was calculated using Student t distribution for two sample hypothesis and two factor analysis of variance (ANOVA) for studies with multiple factors. For both tests a normal distribution was assumed. Generally, we were looking for changes with at least 10% difference from control values. For 95% power and 0.05 significance, this required a minimum of 4-6 animals per group for most of the experiments. In our experience this avoided frequent repetition of experiments with smaller groups especially when a certain degree of biological variability in the response of the animals is anticipated. Comparison were made only between strain-, age- and sex-matched mice and we aimed to use equal numbers of mice in each group to reduce experimental variation, maintain optimal statistical power and avoid statistical artefacts. Experiments aiming at investigating the development of spontaneous skin cancer needed to take into account that the phenotype of the disease depends on several genes acting together with a number of stochastic/environmental factors and thus there is a higher degree of variability (10-20%). For this reason, these experiments were done using sufficiently large groups of mice (8-15 mice per group) to establish unequivocal phenotypic differences and to reach statistically significant results.

Information on the sample size (N-number) of any given experiment is reported in the figure legend.

Replicates

- You should report how often each experiment was performed
- You should include a definition of biological versus technical replication

eLife Sciences Publications, Ltd is a limited liability non-profit non-stock corporation incorporated in the State of Delaware, USA, with company number 5030732, and is registered in the UK with company number FC030576 and branch number BR015634 at the address 1st Floor, 24 Hills Road, Cambridge CB2 1JP | August 2014

- The data obtained should be provided and sufficient information should be provided to indicate the number of independent biological and/or technical replicates
- If you encountered any outliers, you should describe how these were handled
- Criteria for exclusion/inclusion of data should be clearly stated
- High-throughput sequence data should be uploaded before submission, with a private link for reviewers provided (these are available from both GEO and ArrayExpress)

Please outline where this information can be found within the submission (e.g., sections or figure legends), or explain why this information doesn't apply to your submission:

All the experimental data provided are based on biological replicates and are representative of independent experiments. All experiments were reliably reproduced.

No data points were excluded from data sets

High-throughput RNA sequencing data is available from the public repository on the National Center for Biotechnology Information's Sequence Read Archive in raw format (BioProject: PRJNA417372; BioSample accession: SAMN07985450, SAMN07985451, SAMN07985452, SAMN07985453, SAMN07985454, SAMN07985455)

Statistical reporting

- Statistical analysis methods should be described and justified
- Raw data should be presented in figures whenever informative to do so (typically when N per group is less than 10)
- For each experiment, you should identify the statistical tests used, exact values of N, definitions of center, methods of multiple test correction, and dispersion and precision measures (e.g., mean, median, SD, SEM, confidence intervals; and, for the major substantive results, a measure of effect size (e.g., Pearson's r, Cohen's d)
- Report exact p-values wherever possible alongside the summary statistics and 95% confidence intervals. These should be reported for all key questions and not only when the p-value is less than 0.05.

Please outline where this information can be found within the submission (e.g., sections or figure legends), or explain why this information doesn't apply to your submission:

All statistical analysis methods used, the exact values of N, precision measures and multiple test corrections are described in the appropriate figure legends. Statistical analysis methods are additionally described in the 'methods section'.

Raw data is mainly presented throughout.

(For large datasets, or papers with a very large number of statistical tests, you may upload a single table file with tests, Ns, etc., with reference to sections in the manuscript.)

Group allocation

- Indicate how samples were allocated into experimental groups (in the case of clinical studies, please specify allocation to treatment method); if randomization was used, please also state if restricted randomization was applied
- Indicate if masking was used during group allocation, data collection and/or data analysis

Please outline where this information can be found within the submission (e.g., sections or figure legends), or explain why this information doesn't apply to your submission:

For animal work, mice of a particular genotype were randomly selected for experiment from a larger cohort - other than that, no randomization was used. Non-transgenic littermates were used as controls whenever possible or strain-, age- and sex-matched wild-type control animals were purchased from Charles River.

For reporting of tumour development following chemical induced carcinogenesis, back skin and tumours were evaluated by an observer blinded to the experimental groups. Measurements of epidermal thickening was similarly done by an observer blinded to the experimental groups. For most other experiments, the investigators were not blinded to group allocation.

Additional data files ("source data")

- We encourage you to upload relevant additional data files, such as numerical data that are represented as a graph in a figure, or as a summary table
- Where provided, these should be in the most useful format, and they can be uploaded as "Source data" files linked to a main figure or table
- Include model definition files including the full list of parameters used
- Include code used for data analysis (e.g., R, MatLab)
- Avoid stating that data files are "available upon request"

Please indicate the figures or tables for which source data files have been provided:

The source data supporting all the findings of this study are available from the corresponding author. RNA sequencing data supporting Figure 2 is available from the public repository on the National Center for Biotechnology Information's Sequence Read Archive in raw format (BioProject: PRJNA417372; BioSample accession: SAMN07985450, SAMN07985451, SAMN07985452, SAMN07985453, SAMN07985454, SAMN07985455).

eLife Sciences Publications, Ltd is a limited liability non-profit non-stock corporation incorporated in the State of Delaware, USA, with company number 5030732, and is registered in the UK with company number FC030576 and branch number BR015634 at the address 1st Floor, 24 Hills Road, Cambridge CB2 1JP | August 2014
